# Engineered Flock House Virus for Targeted Gene Suppression Through RNAi in Fruit Flies (*Drosophila melanogaster*) *in Vitro* and *in Vivo*

**DOI:** 10.3389/fphys.2018.00805

**Published:** 2018-07-03

**Authors:** Clauvis N. T. Taning, Olivier Christiaens, XiuXia Li, Luc Swevers, Hans Casteels, Martine Maes, Guy Smagghe

**Affiliations:** ^1^Department of Plants and Crops, Faculty of Bioscience Engineering, Ghent University, Ghent, Belgium; ^2^Crop Protection, Flanders Research Institute for Agriculture, Fisheries and Food (ILVO), Merelbeke, Belgium; ^3^Department of Entomology, China Agricultural University, Beijing, China; ^4^Insect Molecular Genetics and Biotechnology Research Group, Institute of Biosciences & Applications, NCSR “Demokritos”, Athens, Greece

**Keywords:** RNAi, Flock House virus, *Drosophila melanogaster*, S2 cells, virus-induced gene silencing, double stranded RNA

## Abstract

RNA interference (RNAi) is a powerful tool to study functional genomics in insects and the potential of using RNAi to suppress crop pests has made outstanding progress. However, the delivery of dsRNA is a challenging step in the development of RNAi bioassays. In this study, we investigated the ability of engineered Flock House virus (FHV) to induce targeted gene suppression through RNAi under *in vitro* and *in vivo* condition. As proxy for fruit flies of agricultural importance, we worked with S2 cells as derived from *Drosophila melanogaster* embryos, and with adult stages of *D. melanogaster.* We found that the expression level for all of the targeted genes were reduced by more than 70% in both the *in vitro* and *in vivo* bioassays. Furthermore, the cell viability and median survival time bioassays demonstrated that the recombinant FHV expressing target gene sequences caused a significantly higher mortality (60–73% and 100%) than the wild type virus (24 and 71%), in both S2 cells and adult insects, respectively. This is the first report showing that a single stranded RNA insect virus such as FHV, can be engineered as an effective *in vitro* and *in vivo* RNAi delivery system. Since FHV infects many insect species, the described method could be exploited to improve the efficiency of dsRNA delivery for RNAi-related studies in both FHV susceptible insect cell lines and live insects that are recalcitrant to the uptake of naked dsRNA.

## Introduction

RNA interference (RNAi), the process that is triggered by double stranded (ds) RNA molecules and results in specific gene silencing through hybridization of processed small RNAs to target mRNAs, has evolved rapidly to a standard technique to evaluate gene function in eukaryotes (Fire et al., 1998; Huvenne and Smagghe, 2010; Zotti et al., 2017). In insects, as many successful experiments were performed, it also became quickly apparent that silencing variability can weaken the applicability of RNAi significantly (Terenius et al., 2011; Scott et al., 2013). For successful gene silencing, efficient cellular uptake of dsRNA is crucial, amongst other factors (Scott et al., 2013; Joga et al., 2016). While naked dsRNA can efficiently be taken up by coleopterans such as, *Diabrotica virgifera* and *Leptinotarsa decemlineata* (Zhu et al., 2011; Bolognesi et al., 2012), specific formulations to enhance the cellular uptake of dsRNA are thought to be necessary for efficiently inducing RNAi in a majority of other insect species (Baum and Roberts, 2014; Joga et al., 2016; Taning et al., 2016). The presence of nucleases in the insect midgut and hemolymph can quickly degrade dsRNA molecules, hence limiting its potential to trigger the RNAi mechanism (Terenius et al., 2011; Allen and Walker, 2012; Christiaens et al., 2014). Furthermore, high pH conditions, as seen in Lepidopterans, which have a strong alkaline pH in their gut, provides a hostile environment for dsRNA. In a comparative study, a much higher stability and tissue penetrance of dsRNA was found for the RNAi-sensitive coleopteran insects than for the RNAi-recalcitrant lepidopterans (Ivashuta et al., 2015). These all imply that the stability of dsRNA in the insect body could be affected by both enzymatic hydrolysis and other elements of the gut environment (Price and Gatehouse, 2008). For protecting and improving cellular uptake of dsRNA in insects, two different approaches have been used that are either based on synthetic nanoparticles (Whyard et al., 2009; Chiu et al., 2013; Das et al., 2015; Taning et al., 2016; Christiaens et al., 2018) or employ engineered micro-organisms (Zhu et al., 2011; Kumar et al., 2013; Taning et al., 2016; Whitten et al., 2016) that produce dsRNA molecules. Among the use of engineered micro-organisms for improved delivery, the possible application of engineered insect viruses for this purpose has received much less attention. Nevertheless, viruses have several attractive properties that make them excellent delivery vehicles for nucleic acids such as, efficiency and specificity of infection and the evolved avoidance of the immune response.

Virus-induced gene silencing (VIGS) is an RNA silencing-based technology that can be exploited to silence genes of interest in insects (Kolliopoulou et al., 2017). Briefly, infection by a virus triggers RNA silencing, an insect innate defense pathway that specifically degrades the viral genome. If the virus is engineered to carry a fragment of an insect gene transcript, RNA silencing would be directed to target this particular endogenous gene. In the past decade, a number of viral vectors have been developed as a powerful reverse genetic tool for the functional characterization of genes in plants (Kumagai et al., 1995; Ruiz et al., 1998; Purkayastha and Dasgupta, 2009; Lange et al., 2013). However, the majority of the published VIGS vectors have a host range limited to some plant species, and very few have been developed for functional genomic studies in insects (Kolliopoulou et al., 2017). Currently, only two insect virus-based RNAi delivery systems have been developed. The baculovirus system based on *Autographa californica* nuclear polyhedrosis virus (AcMNPV) (Huang et al., 2007; Kontogiannatos et al., 2013) and the densovirus system based on *Aedes aegypti* densovirus (AeDNV) (Gu et al., 2011). Briefly, AcMNPV and AeDNV are DNA viruses which have a limited host range (lepidopterans and mosquitoes, respectively) and can be easily manipulated and produced in cell lines. These properties have driven research on these viruses for potential applications in environmentally safe pest control, and as gene transduction and RNAi delivery vectors. However, their specificity to a limited number of insect species is also a drawback in research, since it limits the exploitation of these virus-based RNAi delivery systems for use in many other insects.

In this study, we aimed to engineer Flock House virus (FHV) as a virus-based RNAi delivery system in insects. Since FHV is known to infect and replicate in many other insect species (Selling and Rueckert, 1984; Gallagher and Rueckert, 1988; Swevers et al., 2016), this will provide an ideal delivery system for functional studies in different RNAi recalcitrant insects. FHV belongs to the Nodaviridae family and the Alphanodavirus genus, and was first isolated from the grass grub *Costelytra zealandica* (Coleoptera) in New Zealand (Dearing et al., 1980). FHV has a simple genome organization composed of two positive-sense, single-stranded RNAs packaged by a single capsid into a non-enveloped icosahedral virion (Scotti et al., 1983; Schneemann et al., 1998). RNA1 is 3.1 kb in length and encodes the autonomous viral RNA-dependent RNA polymerase (RdRp, protein A; 112 kDa) (Friesen and Rueckert, 1981; Poch et al., 1989; Price et al., 2000). During FHV replication, a subgenomic RNA3 (0.4 kb) is also synthesized which encodes two proteins, B1 and B2 (Guarino et al., 1984). The function of translated B1 protein is poorly defined, but may be important for maintenance of RNA replication (Ball, 1995), whereas protein B2 is responsible for suppressing Dicer-mediated RNA silencing (Li et al., 2002). Genomic RNA2 (1.4 kb) encodes the viral capsid protein precursor, CP-α (43 kDa), that is later cleaved into 40 kDa (β) and 4 kDa (γ) fragments after proviron assembly (Friesen and Rueckert, 1981; Schneemann et al., 1998). The autonomous ability of the FHV RNA1 to replicate and the robust intracellular genome synthesis and protein expression directed by subgenomic promoters makes FHV an ideal candidate for amplifying heterologous sequences.

Flock House virus has served as a model to study many different aspects of infection by small positive sense RNA viruses such as non-enveloped viral cell entry, RNA viral genome replication, specific packaging of two genome segments, virion assembly and the RNAi-mediated immune response (Venter and Schneemann, 2008; Odegard et al., 2010). While biotechnological applications based on FHV include production of viral-like particles for vaccine developments (Schneemann et al., 2012), its development as a gene transduction or VIGS vector still remains to be realized. For the latter purpose, two strategies can be followed. The first strategy, which is followed in this work, consists of the insertion of foreign gene sequences in RNA1 in such a manner that the functions of RdRp and B2 are not disrupted (Price et al., 2000). The other strategy, which was used to achieve gene transduction in mosquito larvae, is based on the engineering of a defective interfering RNA derived from RNA2 (Dasgupta et al., 2003). For recombinant FHV production, expression vectors for both RNA1 and RNA2 are necessary, in order to provide replication machinery and capsid proteins for transmission, respectively.

**FIGURE 1:**
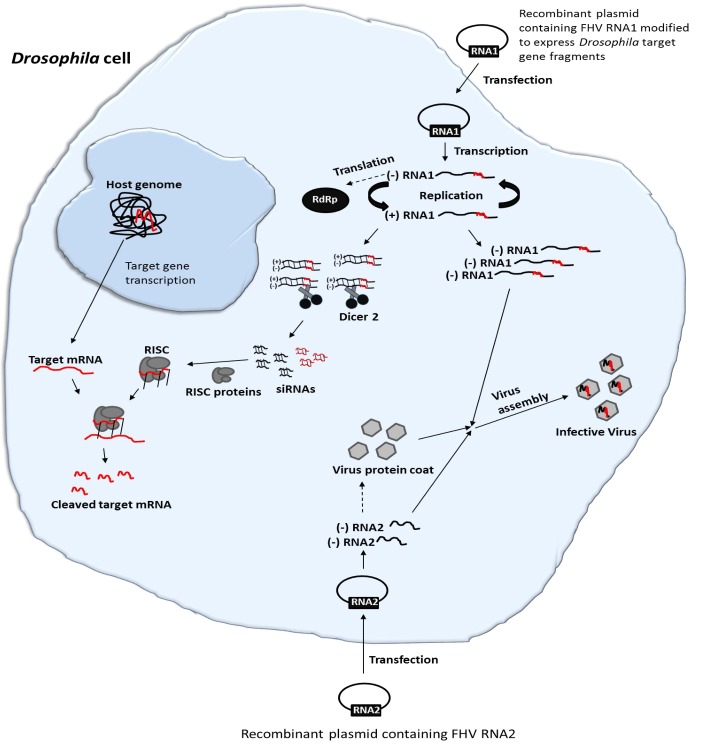
Schematic illustration of FHV-based RNAi delivery in *Drosophila*. Cell transfection and activation of FHV RNA1 and RNA2-based plasmids lead to the expression of the FHV genome. During the replication of FHV RNA1, dsRNAs are formed. Since FHV infection results in production of viral siRNAs, an insertion of target gene sequences in the FHV genome could therefore be employed to deliver specific RNAi effects in infected cells. Furthermore, the system will generate functional recombinant virions that can infect neighboring cells, spreading the RNAi signal.

A FHV plasmid-based system whereby an expression cassette that transcribes RNA1 with precise 5′- and 3′-ends, realized by positioning of the promoter sequence and self-cleaving ribozyme, respectively, can initiate high levels of FHV replication. In the presence of RNA2, the replication system will generate functional virions. Since FHV infection results in production of viral siRNAs (Gammon and Mello, 2015), an insertion of foreign sequences in the FHV genome could therefore be employed to deliver specific RNAi effects in infected cells (**Figure 1**). Herein, we used S2 cells that are derived from embryos of *Drosophila melanogaster* as proxy for fruit flies of economic importance in agriculture, for instance the spotted wing *Drosophila suzukii* but no cell line is available for this important pest. We first developed a recombinant FHV expressing selected *D. melanogaster* target gene sequences and then assessed whether it could replicate and induce targeted gene suppression in *D. melanogaster*. To this end, we first examined whether the engineered FHV plasmids were able to express the recombinant FHV clones in S2 cells. We then showed that the resulting infective recombinant FHV can induce gene silencing *in vitro* in S2 cells and *in vivo* in adult stages of *D. melanogaster*. Overall, our findings show the potential of engineering FHV for VIGS in insects. Furthermore, this study opens a potential avenue for more research into the use of insect viruses for viral vector-mediated silencing of target host genes, and the engineered FHV vector may serve as a powerful molecular tool for functional genomic studies in both FHV susceptible insect cell lines and life insects, which are recalcitrant to the uptake of naked dsRNA.

## Materials and Methods

### Cell Culture

Schneider 2 (S2) cells, as derived from a primary culture of *D. melanogaster* embryos (Schneider, 1972), were maintained at 27°C in InsectXpress culture medium (Lonza) supplemented with 10% Fetal Bovine Serum (FBS) (Sigma-Aldrich) at the Laboratory of Agrozoology, Ghent University, Belgium.

### Insect Culture

*Drosophila melanogaster* adults and larvae were reared on an agar-yeast-cornmeal diet slightly modified from Lebreton et al. (2014) (8 g agar, 60 g corn meal, 60 g brewer’s yeast, 25 g sucrose, 600 ml distilled water and 2.5 g methyl-4-hydroxybenzoate dissolved in 25 ml of 70% ethanol) at standard laboratory conditions of 25°C, 65% relative humidity and under a 12:12 light: dark photoperiod. Prior to the bioassays, female and male adult flies were transferred to new diet tubes for 6 h for egg-laying. The resulting synchronized mixed population of both male and female flies were then used for the bioassays.

### Target Gene Selection

The target genes (**Table 1**) used in this study were selected based on our previous study which reported on their effectiveness in causing mortality to the closely related species, *D. suzukii* (Taning et al., 2016). Target gene sequences for *D. melanogaster* were located in the database of *Drosophila* genes and genome^1^ by BLAST analysis using known query sequences from other insects. The chosen target region from each gene selected was amplified using designed synthetic primers containing restriction sites for AsiSI (GCGATCGC) and BsrGI (TGTACA) flanking both the 5′ and 3′ ends, respectively (**Table 1**). Enhanced green fluorescent protein gene (*eGFP*) was used as a reporter gene in this study. The entire *eGFP* sequence was amplified with synthetic primers including restriction sites for NsiI (ATGCAT) and AsiSI flanking its 5′ and 3′ ends, respectively (**Table 1**).

**Table 1:** Primers for genes used in designing the FHV-based RNAi delivery system.

Target genes	Symbol	Accession	Primer sequence (5′–3′)^∗^	Product size (bp)
Alpha-coatomer protein, isoform A	alpha COP	NM_058047.5	Forward: TGATCGCCTTGTGAAGT	499
			Reverse: GATCGTAGGTGCTGTTCTCCA	
Ribosomal protein S13	RPS13	X91854.1	Forward: GCAGATGATGTCAAGGA	421
			Reverse: ATGTAGGACCCCGCAAGAC	
Vacuolar H[+]-ATPase 26kD E subunit	Vha26	U38198.1	Forward: AGCACCGAAATGGACCT	449
			Reverse: ATTGGCGAACATGCGAATA	
Enhanced Green Fluorescent protein (Reporter)	eGFP	/	Forward: ATGGTGAGCAAGGGCGAGGA	720
			Reverse: TTACTTGTAGAGCTCGTCCA	

*^∗^Restriction enzyme sites are not included in the primer sequence.*

### Plasmid Constructs for the Expression of Both eGFP and Target Gene Sequences

**FIGURE 2:**
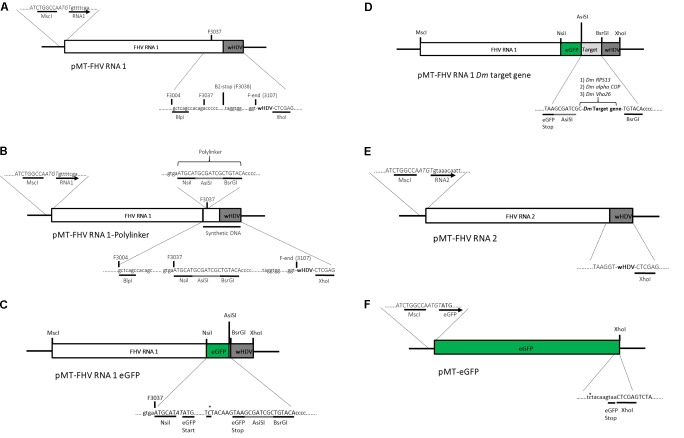
Schematic illustration of FHV RNA1-based plasmid systems for foreign gene expression in *Drosophila* S2 cells. **(A)** Plasmid system expressing the complete wild type FHV-RNA1, **(B)** Plasmid system expressing FHV-RNA1 with a polylinker inserted at position 3037 bp, **(C)** Plasmid system expressing FHV-RNA1 with eGFP inserted into the polylinker between the NsiI and AsiSI restriction sites, **(D)** Plasmid systems expressing FHV-RNA1 with eGFP and a *Drosophila melanogaster* target gene sequence, **(E)** Plasmid system expressing FHV-RNA2, **(F)** Plasmid system expressing only eGFP.

Standard molecular cloning techniques were used unless otherwise stated. A plasmid (pMT/V5-His A) containing the full FHV RNA1 genome and a ribozyme sequence derived from hepatitis delta virus (HDV) attached to its 3′ end was kindly provided by Professor Ronald Van Rij (Radboud Institute for Molecular Life Sciences, Nijmegen, Netherlands) (**Figure 2A**). Based on Maharaj et al. (2014), an insertion site was created at position 3037 bp of the pMT-FHV RNA1 genome for the introduction of the reporter gene (*eGFP*) and subsequently a *D. melanogaster* target gene sequence for dsRNA production during viral replication. First, a polylinker, ATGCATGCGATCGCTGTACA, composed of three restriction sites, NsiI, AsiSI, and BsrGI was inserted into position 3037 bp of the pMT-FHV RNA1 genome (**Figure 2B**). After confirmation by sequencing and restriction digest analysis, *eGFP* was introduced in between NsiI and AsiSI restriction sites to create pMT-FHV RNA1-GFP replicons (**Figure 2C**). Additional expression constructs were generated where *D. melanogaster* target genes (*Vha26*, *RPS13*, and *alpha COP*) were inserted after *eGFP* in between AsiSI and BsrGI restriction sites (**Figure 2D**). The FHV RNA2 sequence tailed at its 3′ end by the HDV ribozyme sequence (**Figure 2E**) was synthesized (by gene synthesis: Thermo Fisher Scientific) and cloned into the vector backbone of the pMT-FHV RNA1 plasmid (plasmid without FHV RNA1). A non-virus positive control construct for eGFP expression was made by replacing the FHV RNA1 genome in the plasmid with *eGFP* (**Figure 2F**). All constructs were sequenced to ensure maintenance of sequence identity and to assess for spurious mutations in the constructs (Supplementary File).

### Transfection of S2 Cells and Virus Detection by Fluorescence Microscopy

S2 cells were transiently transfected with the engineered plasmids using Escort IV (Sigma-Aldrich), according to the manufacturer’s instructions. Briefly, each well of a 6-well plate was filled with 3 × 10^6^ S2 cells and washed twice with serum-free culture medium. 14 μl of the Escort IV was first pre-incubated alone for 30–45 min in serum-free culture medium and then 30 min together with 1.5 μg of each of the pMT-FHV RNA1 plasmids plus 1.5 μg of the pMT-FHV RNA2 plasmid (1:1 ratio). The transfection medium was added to the cells and incubated for 16 h at 27°C. After incubation, the transfection medium was replaced with fresh medium containing serum (10% FBS) and further incubated for 24 h for cell recovery. Following cell recovery, copper sulfate (CuSO_4_) was added to the medium to a final concentration of 700 μM to activate the plasmid promoter to express FHV RNA1 and 2. S2 cells were then observed for eGFP expression at 72 h post-activation using a Nikon Eclipse TS-100 microscope (Melville, NY, United States) and NIS Elements BR 4.11.00 imaging software (Nikon, Melville, NY, United States). The plasmid containing only *eGFP* (pMT-eGFP) was used as positive control for transfection and plasmid activation for eGFP expression, while the plasmid containing the wild type (WT) genome, pMT-FHV RNA1, was used as a negative control (no eGFP expression).

### Detection of FHV Expression by PCR

After confirmation of eGFP expression by imaging, cells obtained from each treatment were lysed and total RNA was extracted using the RNeasy Mini Kit (Qiagen). After DNase I treatment (Ambion) to remove residual genomic and plasmid DNA, the RNA was quantified using a NanoDrop ND-1000 (Thermo Scientific) and verified by 1.5% agarose gel electrophoresis. Total RNA (1 μg) was reverse transcribed using the SuperScript IV kit (Invitrogen) according to manufacturer’s instructions. The resulting complementary DNA (cDNA) was used as a template in a PCR reaction for the detection of FHV using designed primers (**Table 2**). The primers used for FHV detection were designed to detect the negative strand of the virus, so as to further confirm for virus replication. The PCR reactions included 0.2 μl of Taq DNA polymerase (Invitrogen), 2 μl of 10x PCR buffer (Invitrogen), 0.6 μl of 10 μM forward primer (Invitrogen), 0.6 μl of 10 μM of reverse primer (Invitrogen), 0.6 μl of 50 mM MgCl_2_, 0.6 μl of 10 mM dNTPs, 15 μl of nuclease-free water and 0.9 μl of cDNA, in a total volume of 20.5 μl. The amplification conditions were 2 min at 94°C followed by 33 cycles of 30 s at 94°C, 30 s at 60°C and 45 s at 72°C, and then 10 min at 72°C and infinity at 10°C.

**Table 2:** Primers used for PCR detection of FHV RNA1 and 2.

Target genes	Primer sequence (5′–3′)	Product size (bp)
FHV RNA1	Forward: GTTGGGACGGTTTATTCAGC	400
	Reverse: ATCGGTATGGGACACAAGGA	
FHV RNA2	Forward: ATCAAGAGGTGGCGAGTCAT	500
	Reverse: GCATTTACCCAACGTCGAAC	

### Virus Amplification

While some cells were collected for FHV detection by PCR, the infectious viral particles were harvested from the remaining cells and supernatant (72 h post-transfection). First, the cells from each treatment were centrifuged at 1000 rpm for 5 min and then 90% of the medium was taken out. The cells and residual medium were subjected to two cycles of freeze-thawing and later centrifugation at 8000 rpm for 10 min to remove cell debris. The unpurified infectious virus supernatant was used to infect virus-free S2 cells and the cells were then incubated for 72 h at 27°C (Supplementary File). After incubation, the infectious virus was extracted from the cells as described above and the process was repeated three times with the aim of increasing the viral load. Based on preliminary experiments, concentrating the viral load three times was just enough to avoid more than 90% S2 cell mortality after 72 h. The supernatant containing the infective virus, obtained from the third repeat, was used to infect S2 cells and adult *D. melanogaster* in the *in vivo* and *in vitro* bioassays, respectively. This step was repeated four times for each separate biological repeat per treatment. Prior to the bioassays, qRT-PCR was used to confirm that the viral titer was the same between the gene targeting and non-gene targeting FHV inoculums (Supplementary File).

### Cell Viability Assays

For these assays, 100 μl of the unpurified viral supernatant (for each treatment) was added to each well of a 6-well plate filled with 3 × 10^6^ S2 cells and then incubated at 27°C. The infected cells were observed daily under a light microscope. After 72 h, a time point where a clear difference in cell growth could be visually observed between the different treatments, live and dead (stained with 0.4% Trypan blue) cells were enumerated manually under a light microscopy (10× magnification), using a Neubauer hemocytometer according to (Decombel et al., 2004; Chan et al., 2015). Cell viability was calculated as the ratio of live cells to death cells in the total cell population. This experiment was repeated four times for each separate biological repeat per treatment.

### Survival Bioassay

Survival bioassays were performed by infecting *D. melanogaster* adults with the engineered virus and then monitoring their survival over time. Three to four-day-old *D. melanogaster* adult flies were anesthetized with diethyl ether for 2 min, immobilized in a 1.5% agarose plate and injected with the unpurified virus supernatant. A volume of 100 nL of the treatments (RNA1 RS13, RNA1 Vha26, RNA1 alpha COP) and controls (RNA1 eGFP, RNA1, no virus), obtained as described above, was injected into the hemolymph using a microinjector (FemtoJet, Eppendorf) and needles prepared with glass capillary tubes. At least 14 to17 adult flies were injected per treatment and this was repeated four times to give a total number of 61 adults injected per treatment. After injection, the flies were allowed to recover for 10 min in a horizontally placed 50 ml tube, and then transferred into 50 ml tubes containing 10 ml of diet and incubated at 25°C and 65% RH. The flies were evaluated for mortality every day for 15–20 days. Four surviving insects per treatment were taken on the 4th day (the day with the highest observed mortality), pooled and homogenized in RLT buffer (Qiagen) + β-mercapto ethanol for RNA extraction, and stored in the buffer at -80°C until further purification and transcript analysis. This was repeated for each replication of the bioassay.

### Reverse Transcription Quantitative PCR (RT-qPCR)

Total RNA was extracted using the RNeasy Mini Kit (Qiagen). After DNase I treatment (Ambion) to remove residual genomic and/or plasmid DNA, the RNA was quantified using a NanoDrop ND-1000 (Thermo Scientific) and verified by 1.5% agarose gel electrophoresis. Total RNA (1 μg) was reverse transcribed using the SuperScript IV kit (Invitrogen) according to manufacturer’s instructions. Real time quantitative PCR was performed in the CFX 96TM real-time system and the CFX manager software (Bio-Rad). The primers used in the analysis (**Table 3**) were validated with a standard curve based on a serial dilution of cDNA to determine the primer annealing efficiency and a melting curve analysis with temperature range from 60 to 95°C. The reaction included 10 μl of SYBR green Supermix (Bio-Rad), 0.4 μl of 10 μM forward primer (Invitrogen), 0.4 μl of 10 μM of reverse primer (Invitrogen), 8.2 μl of nuclease-free water and 30 ng of cDNA, in a total volume of 20 μl. The amplification conditions were 3 min at 95°C followed by 39 cycles of 10 s at 95°C and 30 s at 60°C. The reactions were set-up in 96-well format Microseal PCR plates (Bio-Rad) in triplicates. A fluorescence reading determined the extension of amplification at the end of each cycle and each experiment was repeated four times using samples from independent treatments. The endogenous control gene, alpha-tubulin at 84B (*αTub84B*) was used for normalization of the data. The relative amounts of the target gene transcripts in the S2 cell samples with the engineered FHV containing the *D. melanogaster* target gene specific sequence were normalized to the endogenous reference gene by the equation ratio 2^-ΔΔC_t_^ (Livak and Schmittgen, 2001). Appropriate controls, no-template control and no reverse transcriptase control, were also included in the assay.

**Table 3:** Primers used in quantitative RT PCR.

Target genes	Symbol	Accession	Primer sequence (5′–3′)	Product size (bp)
Alpha-coatomer protein, isoform A	alpha COP	NM_058047.5	Forward: GGGTCAGAGCATCATTGCTT	100
			Reverse: CTCCAGAGCGAGTCCAAATC	
Ribosomal protein S13	RPS13	X91854.1	Forward: CCGTCTGATTCTGGTCGAGT	99
			Reverse: GCAGTGCTCGACTCGTATTTC	
Vacuolar H[+]-ATPase 26kD E subunit	Vha26	U38198.1	Forward: GCACGCGACACTTAATACCC	99
			Reverse: GTGAAAGCTGCACTTGATGG	
Alpha-tubulin at 84B	αTub84B	NM_057424.4	Forward: TGTCGCGTGTGAAACACTTC	96
			Reverse: AGCAGGCGTTTCCAATCTG	

### Statistical Analysis

Cell viability data between the treated groups was tested using ANOVA followed by Bonferroni’s multi comparison tests. Survival data from treated *D. melanogaster* adults was analyzed according to the Kaplan–Meier method (Kaplan and Meier, 1958). The Gehan–Breslow–Wilcoxon and log-rank (Mantel–Cox) tests were used to compare the statistical significance (*p* < 0.05) between the datasets (controls and treatments). The Gehan–Breslow–Wilcoxon test measures more at early time points, while the log-rank (Mantel–Cox) test measures equally at all time points. The analyses were performed using GraphPad Prism v5.0 software (GraphPad, La Jolla, CA, United States). For the qRT-PCR analysis, the differences between groups were calculated by an unpaired *t*-test (*p* < 0.05) and performed in qBase+ software.

## Results

### Organization and Generation of Recombinant FHV Expressing eGFP as a Reporter Gene

**FIGURE 3:**
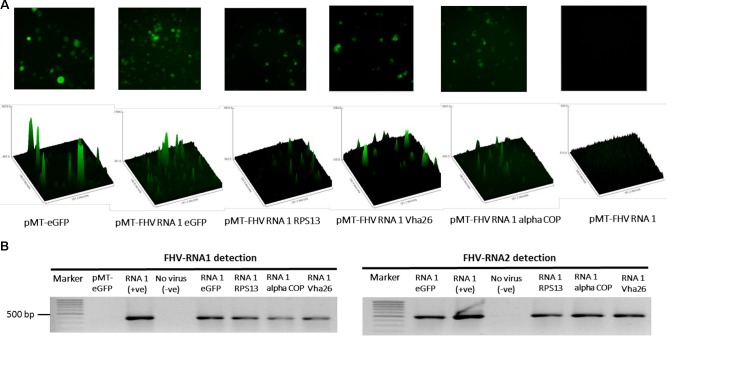
Detection of recombinant FHV RNA1 by fluorescence and PCR. **(A)** Fluorescence microscopy analysis of S2 cells expressing the engineered FHV plasmids, containing an eGFP construct, indicating successful expression of the inserted gene. The FHV RNA1 plasmid, not containing an eGFP fragment was used as negative control, while the plasmid expressing only eGFP without FHV was used as a positive control for eGFP expression. Nikon Eclipse TS-100 microscope and NIS Elements BR 4.11.00 imaging software (Nikon, Melville, NY, United States) were used for the microscopy analysis, **(B)** PCR detection of FHV RNA1 and RNA2 expression.

The modified FHV vectors for targeted gene suppression in *D. melanogaster* were designed by inserting a reporter gene (*eGFP*) and *D. melanogaster* target gene sequences under the control of the B2 subgenomic promoter as shown schematically in **Figure 2D**. More specifically, the insertion occurs after the critical residues necessary for the functioning of the B2 protein (Chao et al., 2005). This design results in the expression of eGFP, which provides a robust marker for confirming the expression of the recombinant vectors in the transfected cells. Direct visualization of green fluorescence in S2 cells at 72 h post-transfection confirmed the expression of the recombinant FHV (**Figure 3A**). As to be expected, eGFP fluorescence was not observed in the cells expressing the wild type FHV genome (pMT-FHV RNA1). However, we could confirm the presence of FHV by PCR in wild type FHV transfected cells which showed no fluorescence (**Figure 3B**).

### Recombinant FHV Can Induce Targeted Gene Suppression and Mortality in *Drosophila* S2 Cells

**FIGURE 4:**
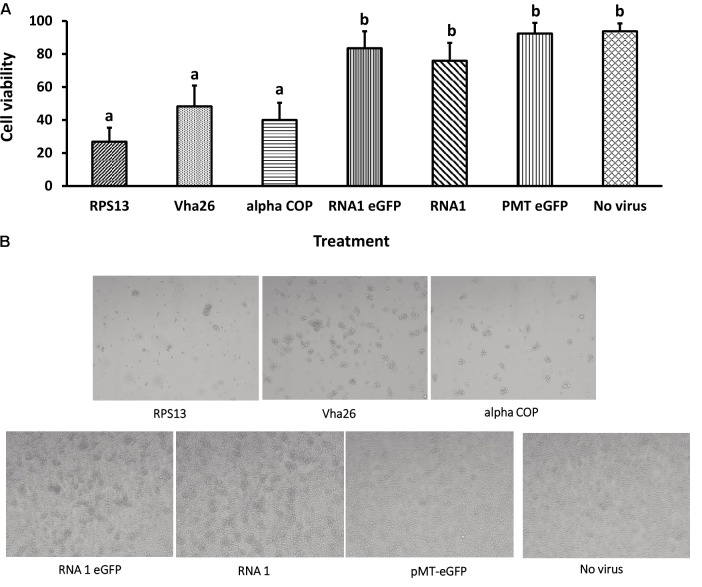
Cell viability at 72 h post-infection with a virus supernatant containing either the wild type FHV (RNA1), either of the four recombinant FHV (RPS13, Vha26, alpha COP, and RNA1eGFP), or No virus controls (pMT eGFP and No virus). **(A)** Cell viability post-infection. Bars represent the mean ± standard error. Different letters indicate statistically significant differences (*p* < 0.05). **(B)** Images showing cell viability at 72 h post-infection.

**FIGURE 5:**
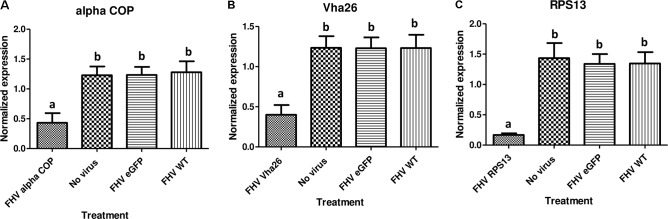
Target gene knockdown: **(A)** *alpha COP*, **(B)** *Vha26*, and **(C)** *RpS13* in *D. melanogaster* S2 cells at 72 h post-infection with the respective recombinant FHV (FHV alpha COP, FHV Vha26 and FHV RPS13) compared to the controls (No virus, wild type FHV and FHV eGFP). Bars represent the mean ± standard error. Different letters indicate statistically significant differences (*p* < 0.05).

With the aim of evaluating the potential bioactivity of the infectious recombinant FHV, the infective virions were harvested from the cells and used to inoculate virus-free S2 cells. At 72 h post-inoculation, a significant decrease (*p* < 0.05) in cell viability was observed for all the samples infected with the gene targeting recombinant virus, RPS13 (27 ± 9%), Vha26 (48 ± 13%), alpha COP (40 ± 10%) compared to the non-gene targeting controls, RNA1 eGFP (83 ± 10%), RNA1 (76 ± 11%), pMT eGFP (92 ± 6%), and No virus (94 ± 5%) (**Figures 4A,B**). Next, we verified whether the observed significant decrease in cell viability for the FHV target gene treatments was linked to the silencing of the targeted genes. To this end, qRT-PCR was performed on samples collected from the infected cells. The transcript level for RPS13 in the FHV RPS13 infected cells was reduced to 13 ± 3% compared to its transcript level in the FHV eGFP infected cells (**Figure 5**). Similarly, for the FHV Vha26- and FHV alpha COP-infected cells, the transcript levels for Vha26 and alpha COP were reduced to 33 ± 12% and 35 ± 16%, respectively, compared to their transcript levels in the FHV eGFP control (**Figure 5**). No significant difference (*p* > 0.05) in expression of the targeted genes was observed between the controls, FHV eGFP, FHV WT (wild type) and No virus.

### Recombinant FHV Can Induce Targeted Gene Suppression and Mortality in Adult Fruit Flies (*Drosophila melanogaster*)

**FIGURE 6:**
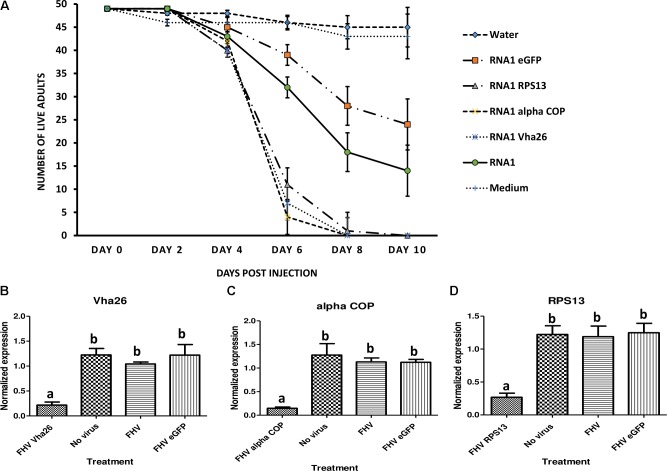
Effect of virus induced gene silencing (VIGS) in *Drosophila melanogaster* flies, using a designed FHV-based RNAi delivery system. **(A)** Survival of adult flies over 10 days post-injection with a virus supernatant containing either the wild type FHV (RNA1), either of the four recombinant FHV (RNA1 RPS13, RNA1 Vha26, RNA1 alpha COP, and RNA1eGFP), water or S2 cell culture medium. Inhibition of the expression of the target genes: **(B)** *Vha26*, **(C)** *alpha COP* and **(D)** *RpS13* in *D. melanogaster* adults at 4 days post-infection with the respective recombinant FHV (FHV Vha26, FHV alpha COP, and FHV RPS13) compared to the controls (No virus, wild type FHV, and FHV eGFP). Bars represent the mean ± standard error. Different letters indicate statistically significant differences (*p* < 0.05).

Once the bioactivity of the recombinant FHV was confirmed *in vitro* in S2 cells, we next aimed to verify whether similar results could be obtained *in vivo* in live insects. To this end, *D. melanogaster* adult flies were infected by microinjection of FHV into the hemocoel and observed daily. Interestingly, between days 4 and 6 post-infection, a big difference could be observed in the survival rates between the groups treated with the recombinant virus expressing the target gene sequences (*RPS13*, *Vha26*, and *alpha COP*) compared to the control groups (FHV eGFP, FHV WT, Medium and Water) (**Figure 6A**). By 10 days post-infection, none of the flies infected with the recombinant virus expressing the target genes survived (0%) in contrary to the control groups, where a significant proportion of the flies survived; FHV eGFP (49%), FHV WT (29%), Medium (88%), Water (92%) (**Figure 6A**). To determine whether this observed difference in mortality between the recombinant FHV target genes treated groups and the control groups was linked to target gene silencing, samples were collected on the 4th day (day with first high mortality in test groups) for gene expression analysis. For the FHV alpha COP-treated insects, a significant decrease (*p* < 0.05) in the transcript level for alpha COP (11 ± 1%) compared to the FHV eGFP-treated control was observed (**Figure 6C**). Similarly, for FHV Vha26 and FHV RPS12-infected insects, the transcript levels for Vha26 (18 ± 5%) and RPS13 (22 ± 2%) were significantly (*p* < 0.05) lower when compared to the control FHV eGFP-infected insects (**Figures 6B,D**). No significant difference (*p* > 0.05) in expression of the targeted genes was observed between the controls, FHV eGFP, FHV WT and No virus (Medium)-treated insects (**Figure 6**).

## Discussion

In this study, we hypothesized that engineering FHV to express *D. melanogaster* target gene sequences could lead to VIGS when *D. melanogaster* cells are infected by recombinant clones of the virus. The resulting findings of this study have three important implications. First, we show that a single stranded RNA insect virus can be engineered as a virus-based delivery system, for both *in vitro* and *in vivo* RNA silencing in an insect. Second, FHV is known to infect a wide range of insect species, hence this system could be easily adapted for RNAi- related studies in RNAi-recalcitrant insect species. Lastly, by using RNAi mediated by the recombinant FHV, we have shown that targeting essential genes such as *RPS13*, *alpha COP*, and *Vha26* causes cell mortality, which in turn leads to the death of the insect.

### Generation of Infective Recombinant Virions

In an efficient process, FHV RNA1 combines the properties of a message for an RNA replicase subunit with those of a template for replication by the same enzyme, to specifically direct its own replication in the cytoplasm of appropriate cells. To reconstruct this autonomous RNA replication system from cDNA clones, we based our strategy on Maharaj et al. (2014), where we inserted either *eGFP* alone or *eGFP* and one *D. melanogaster* target gene sequence (*RPS13*, *alpha COP* or *Vha26*) at position 3032 bp of FHV RNA1. Through a CuSO_4_ inducible pMT vector system, primary transcripts of FHV RNA1 were expressed in the cytoplasm of the *Drosophila* S2 cells. These transcripts were designed to undergo ribozyme-mediated autolysis to generate competent templates for self-directed RNA replication. Similar to FHV RNA1, FHV RNA2 transcripts were also designed to undergo ribozyme-mediated autolysis to generate competent templates for capsid protein expression. This was done in accordance with previous studies which have shown that minimizing terminal extensions at the 3′end of the FHV primary transcript is critical in generating RNA molecules which can replicate (Ball, 1995). Using eGFP as a convenient reporter, we demonstrated that all of the recombinant FHV RNA1 transcripts with an eGFP open reading frame (ORF) were expressed, as evidenced by green fluorescence emitted by the cells. Additionally, the replication of the virus was confirmed by the detection of the reverse genome of the virus. These findings are in line with previous studies, which have also demonstrated successful FHV replication and eGFP expression, by using a similar strategy as described in this study (Cho and Dreher, 2006; Zhou et al., 2015; Zhou and Kearney, 2017). Nevertheless, eGFP fluorescence was only used in this study for detecting the expression of the virus. We were more interested in obtaining infective virions which could infect and replicate in *Drosophila* cells. To this regard, we prepared an unpurified virus supernatant through cycles of freeze-thawing and finally centrifugation. This virus supernatant was used to infect virus-free cells, which were then verified after 3 days for the presence of the virus through eGFP fluorescence and transcript detection (see Supplementary File). The observation of fluorescence and detection of the reverse genome of the virus, confirmed its replication in the newly infected cells.

### FHV-Based RNAi Delivery System Is Efficient *in Vitro*

Besides the infectivity of the virus, we were also interested to know if the recombinant virus could induce targeted gene suppression in S2 cells. To this regard, we infected virus-free S2 cells with the unpurified virus supernatant and then evaluated the transcript level of the targeted genes when a visible decrease in cell viability was observed. Preliminary experiments had indicated that after concentrating and infecting new virus-free cells successively three times, the resulting virus supernatant would cause less than 100% mortality after 3 days in newly infected cells. This data was vital for the planning on when to evaluate both cell viability and the transcript levels of the targeted genes in the surviving cells. Our results indicated a significant decrease in cell viability for all of the recombinant virus-infected cells, expressing *D. melanogaster* target gene sequences in comparison to the controls, which consisted of the recombinant virus expressing only eGFP and the wild type virus. Furthermore, transcript analysis of the treated samples indicated that the mRNA levels for the targeted genes significantly decreased in the recombinant virus-treated samples in comparison to the controls. The correlation of a decrease in cell viability to the decrease in target gene transcripts could be explained by the essential role played by the expression products of these genes in the cell. Three target genes, namely *alpha COP*, *RPS13*, and *Vha26*, chosen based on their essential functions, making them good RNAi targets (Taning et al., 2016), were used in this study to evaluate the designed FHV-based RNAi delivery system. *Alpha COP* codes for the subunit of the cytosolic coatomer protein complex that binds to dilysine motifs and reversibly associates with Golgi non-clathrin-coated vesicles, which further mediate biosynthetic protein transport from the endoplasmic reticulum (ER), via the Golgi up to the trans Golgi network (Kitazawa et al., 2012). *Vha26* codes for the subunit of the peripheral V1 complex of vacuolar ATPase essential for assembly or catalytic function. V-ATPase is responsible for acidifying a variety of intracellular compartments in eukaryotic cells (Dow, 1999). *RPS13* codes for a constituent of the small ribosomal subunit. Ribosomes translate all mRNAs produced from nuclear genes and perform the majority of cellular protein synthesis (Alonso and Santarén, 2006). These target genes all play essential roles in the cell, explaining the decrease in cell viability observed when their transcripts levels are significantly reduced. By using a mosquito recombinant densovirus RNAi-based system, Gu et al. (2011) also reported that a 90% decrease in the expression of V-ATPase in C6/C36 cells led to increased cell mortality. However, in this case RNAi was triggered after expression of a short RNA hairpin by an RNA polymerase III promoter, which is predicted to be processed to a single siRNA by Dicer. In the FHV system, on the other hand, many different siRNAs are expected to be produced from the targeted gene region during RNA1 replication. Whether this results in more efficient gene silencing may require a direct comparison of both VIGS systems.

### FHV-Based Delivery RNAi System Is Efficient *in Vivo*

Once the ability of the virus-based RNAi delivery system was confirmed *in vitro* in cells, we proceeded to evaluate its efficiency *in vivo* in adult *D. melanogaster* flies. We used a simple bioassay set up, where we injected the flies with the same batch of unpurified virus supernatant used in the cell bioassays, and then evaluated for target gene silencing at the first signs of high mortality. On day 4 after infecting the flies with the virus, we observed a slight increase in insect mortality. This was, however, not significantly different between the test groups (insects treated with the recombinant virus expressing the target genes) and the control groups (treated with either the recombinant virus expressing only eGFP or the wild type virus). Interestingly, from day 6 till day 10, we observed a significant difference in insect mortality between the test groups and the control groups. Samples collected on day 4 for transcript analysis, exhibited over 70% reduction in the transcript level for the target genes, explaining the significant increase in mortality observed in these test groups compared to the controls.

This thus confirmed the observation from the survival time bioassays, demonstrating that the recombinant FHV expressing the target genes caused more serious pathogenic effects than the wild type virus. A possible limitation for the use of the designed FHV-based RNAi delivery system for functional genomics in live insects could arise from the fact that the FHV will eventually cause mortality in the insect, as it multiplies. This will make it difficult to study a phenotype other than mortality, which arises at a further point in the development of the insect, particularly for insects with long life cycles. Our results in this study indicated that up to 30% of the adult *Drosophila* flies survived for more than 13 days following infection with the wild type FHV. Therefore, the FHV-based delivery system will only be practical if the expected phenotype arises before the virus causes mortality in the insect in question. A possible solution to decrease insect mortality and improve the FHV-based delivery system will be the use of a mild form of FHV (for instance, through altered expression or mutations of the B2 protein; Han et al., 2011; van Cleef et al., 2014), which causes less pathogenic effects in its host, to construct the delivery system. Picorna-like viruses, such as iflaviruses and dicistroviruses (to which FHV belongs), have been reported to often occur as quasi-species in which multiple viral forms complement each other to support infection (Ojosnegros et al., 2011). In this quasi-species population, mild variants/mutants are present and can be selected for further modification and production in cells lines, given appropriate genetic methods. Once this mild recombinant virus infects the cell, the difference in pathogenic effect with the mild wild type-infected cell will be determined by the expected phenotypic effect from target gene silencing. In such case, recombinant FHV could trigger specific gene silencing and concomitant phenotypes in the relative absence of non-specific effects due to viral replication. In another study, where recombinant BmNPV was used to deliver dsRNA targeting a juvenile hormone esterase-related (JHER) gene in the corn borer, *Sesamia nonagrioides*, many non-specific pathogenic effects associated with disrupted metamorphosis were observed in both the tests and controls (Kontogiannatos et al., 2013), despite the restricted host specificity of BmNPV (Maeda et al., 1993). A similar solution as discussed above to the pathogenic effect of the wild type was either the isolation or generation of incapacitated baculoviruses [that is baculoviruses that are deficient for an essential gene in the infection cycle, such as *ie-1* or *lef-8* (Efrose et al., 2010; Ioannidis et al., 2016)]. In this way, the incapacitated baculoviruses can enter target cells and initiate early gene expression without progressing to the late phase of the infection cycle and cell lysis.

In this study, the unpurified virus was used for both the *in vitro* (cell) and *in vivo* (live flies) bioassays. This is not an uncommon source of inoculum for infection. In fact, Gu et al. (2011) reported that when *Aedes albopictus* larvae were infected with unpurified recombinant *Aedes aegypti* densovirus (AeDNV), the expression of V-ATPase was downregulated by nearly 70% compared to controls. Virus-based expression systems are particularly useful for their easy manipulation, higher transfection efficiency, longer-term expression, and more persistent silencing effects *in vivo* (Sliva and Schnierle, 2010). Further studies to improve the use of this FHV-based RNAi delivery system will involve evaluating the infectivity of the virus through the oral route. Although injection, as used in this study, is one of the most commonly used delivery methods for *in vivo* delivery, it is technically demanding. Hence, optimizing an oral delivery method will facilitate the usage of this novel virus-based RNAi delivery system.

While FHV has been reported to orally infect *Drosophila*, high concentrations are needed that typically are achieved after purification of the virions (Ferreira et al., 2014). Because of its relatively difficult set-up that requires ultracentrifugation (Schneemann and Marshall, 1998), the approach of purified virions was not pursued in this work. The concentration of virions [10^8^–10^10^ median tissue culture infective dose (TICD_50_)/ml] that is required for oral infection of *Drosophila melanogaster* and mosquito larvae (Dasgupta et al., 2003; Ferreira et al., 2014) seems too high to have practical applications in pest control and the oral approach therefore needs considerable optimization with respect to infectivity efficiency. Other RNA viruses, such as *Drosophila* C virus or Nora virus, that naturally infect *Drosophila* through the oral route (Habayeb et al., 2009; Ferreira et al., 2014; Lee and Vilcinskas, 2017), may be more suitable candidates for VIGS in pest control, although a reverse genetics system still needs to be developed. For the use of recombinant viruses in the field, however, safety issues need to be taken into account, such as the mutation rate of the viral genome (Elena and Sanjuán, 2005) and its host range (Kolliopoulou et al., 2017).

Nevertheless, as reported in this study, this novel FHV-based RNAi delivery system can be exploited, for functional genomic studies in *Drosophila*, for instance the agriculturally important spotted wing *D. suzukii*. Furthermore, the ability of FHV to infect many insect species, gives this developed virus-based delivery system a unique ability to be broadly used for functional genomic studies in different RNAi-recalcitrant insect species.

## Author Contributions

CT, LS, OC, and GS conceived and designed the research. CT and XL conducted the experiments. HC, MM, and GS contributed new reagents and/or analytical tools. CT analyzed the data. CT, OC, LS, and GS wrote the manuscript. All authors assisted in the critical follow up of the work, read and approved the manuscript.

## Conflict of Interest Statement

The authors declare that the research was conducted in the absence of any commercial or financial relationships that could be construed as a potential conflict of interest.

## Footnotes

1 http://flybase.org/
