# Supplementary material for: Engineered Flock House Virus for Targeted Gene Suppression Through RNAi in Fruit Flies (Drosophila melanogaster) in Vitro and in Vivo
Source: Front Physiol. 2018 Jul 3;9:805. doi: 10.3389/fphys.2018.00805 (PMC6037854; doi:10.3389/fphys.2018.00805)
Supplement: Supplementary file 1 [file Data_Sheet_1.DOCX]

Supplementary Material

**Engineered Flock House Virus for targeted gene suppression through RNA interference in *Drosophila* *melanogaster***

**Clauvis Nji Tizi Taning^1,4*£^, Olivier Christiaens^1*£^, XiuXia Li^2^ , Luc Swevers^3^, Hans Casteels^4^, Martine Maes^4^ , Guy Smagghe^1*^**

^1^Department of Crop Protection, Faculty of Bioscience Engineering, Ghent University, B-9000 Ghent, Belgium

^2^Department of Entomology, China Agricultural University, Beijing, 100193, P. R. China

^3^Insect Molecular Genetics and Biotechnology Research Group, Institute of Biosciences & Applications, NCSR “Demokritos”, Aghia Paraskevi, Athens, Greece

^4^Crop Protection, Flanders Research Institute for Agriculture, Fisheries and Food (ILVO), Burgermeester Van Gansberghelaan 96, 9820 Merelbeke, Belgium

**^£^ Equally contributed**

*** Correspondence:**

Clauvis N. T. Taning

tiziclauvis.taningnji@ugent.be

Olivier Christiaens

Olchrist.christiaens@ugent.be

Guy Smagghe

guy.smagghe@ugent.be

# Supplementary Data 1

Sequence data for pMT plasmid expressing the recombinant Flock House Virus (FHV) RNA1, modified to express enhanced green fluorescent protein (eGFP)

**>pMT-FHV RNA1 eGFP**

tggtgcccgatgtgactagctctttgctgcaggccgtcctatcctctggttccgataagagacccagaactccggccccccaccgcccaccgccacccccatacatatgtggtacgcaagtaagagtgcctgcgcatgccccatgtgccccaccaagagctttgcatcccatacaagtccccaaagtggagaaccgaaccaattcttcgcgggcagaacaaaagcttctgcacacgtctccactcgaatttggagccggccggcgtgtgcaaaagaggtgaatcgaacgaaagacccgtgtgtaaagccgcgtttccaaaatgtataaaaccgagagcatctggccaatgtgttttcgaaacaaataaaacagaaaagcgaacctaaacaatgactctaaaagttattcttggagaacaccagatcacccgaactgaattgttagtcgggattgcaaccgtatctgggtgcggtgccgtagtgtactgcatatccaagttctggggctatggggcaattgcgccctatcctcagagtggagggaaccgagttacacgcgcattgcaacgggctgtcattgacaaaacgaagaccccgatagagacacgtttctatccgcttgacagcctgcgtaccgtgacgcctaagcgtgtcgcagacaacgggcacgccgtttcaggggccgtacgtgatgccgcacgtcgtttgatcgacgagtccatcacggccgttggaggatccaaatttgaggtcaaccccaacccaaactcaagcactggactgcgaaaccatttccacttcgccgtcggtgatttggcacaagatttccgtaatgacacacctgcggatgatgccttcatcgtcggtgttgatgttgattattatgtcaccgagcctgatgtgcttttagagcacatgcgtccagtagtgttacacacctttaacccgaagaaagtgagcggttttgatgctgactcaccattcaccattaagaacaacttggttgaatataaggttagcggtggagcagcatgggtccatccagtttgggattggtgcgaagctggtgagtttatcgctagcagagtccgtaccagctggaaggagtggtttttacaactaccactgcgaatgattggtttggagaaagttggctatcataaaatccatcattgtagaccgtggactgattgtccagatcgtgcacttgtctacactataccgcaatatgtcatttggcggtttaattggattgataccgaactacacgtgcgaaaactgaaacggattgaataccaggacgaaaccaaacctggttggaacagattggagtatgtgaccgacaagaatgaactgctggtttccatcggtcgagaaggggagcatgctcagattactatcgagaaagaaaagttggatatgctctcgggattatccgccacccaatctgtcaacgctaggcttatcggtatgggacacaaggacccgcaatacacatccatgattgtccagtattatactggcaagaaggtagtgtcaccaattagtccaactgtgtataaacctacaatgccacgcgtccattggccagtaaccagtgacgcagatgtaccagaagtgagcgcgcgccaatacacactgcctatcgtgagtgactgtatgatgatgccaatgatcaagcgctgggaaacaatgtctgaatcaattgaacgtagggtgacttttgtcgccaatgataagaaaccaagcgacagaatcgccaaaatagccgaaacgtttgttaaattgatgaatgggccattcaaagatcttgaccctttgtcgattgaagaaacgattgaacggctgaataaaccgtcccaacaattacaacttagggcggttttcgaaatgattggagttgaacctcgtcaattgattgagtcgttcaacaagaacgaacctggaatgaaatctagccggataatatccggttttccagacatacttttcatcttgaaagtttccagatacaccttagcgtattcggatatagttctacatgccgaacacaatgaacattggtattaccccgggcggaacccgactgagatcgccgacggtgtttgtgagtttgttagtgactgtgatgctgaagtcatagaaactgacttctccaacctcgatggcagggtttccagctggatgcaaagaaacatcgcccaaaaggccatggttcaagcattccgcccagaatacagagatgagatcatttcattcatggacacgataatcaattgtccagctaaagctaaacgctttggtttccgatatgagcctggtgtaggcgttaaaagtggaagtccaacaaccacgccacataacacccaatacaatggatgtgtcgaatttacagctctgacctttgagcatcctgatgctgaacctgaagatttgttccgtttaatcggaccgaagtgcggtgatgatggtctttcccgggctatcattcaaaaatcaattaatcgcgctgccaagtgtttcggcctcgaactcaaagttgaacgatacaatccagagataggtctttgtttcctgtctcgtgtatttgtggacccgctcgcaactacgaccacaattcaagacccactgcgtactctgcgaaaactacatcttacaacaagagatccaacgataccattagctgatgcggcttgcgaccgtgtcgaaggctatctctgtaccgatgcgcttactccgttaatttcggattattgcaaaatggtactacgactctacgggcccactgcttcaactgagcaggtgagaaaccaacgtagaagccggaataaagagaagccctactggttgacttgtgacggatcatggccacagcatccgcaagacgcccatttgatgaagcaggttttaatcaaacgtacagccattgacgaagatcaggtcgatgcactcattgggcgttttgccgcaatgaaggatgtctgggagaaaattacacatgacagcgaggagagcgccgctgcgtgtacgtttgatgaagacggcgttgcgccgaactccgtggacgaatcgttaccaatgttaaacgatgccaagcaaactcgcgctaatccaggaacttcccgaccgcattcaaacggcggtggaagcagccatgggaatgagctaccaagacgcaccgaacaacgtgcgcagggacctcgacaacctgcacgcttgcctaaacaaggcaaaactaacggtaagtcggatggtaacatcactgctggagaaacccagcgtggtggcatacctagagggaaaggcccccgaggaggcaaaaccaacactcgaagaacgcctccgaaagctggagctcagccacagccttccaacaaccggaagtgaatgcatatggtgagcaagggcgaggagctgttcaccggggtggtgcccatcctggtcgagctggacggcgacgtgaacggccacaagttcagcgtgtccggcgagggcgagggcgatgccacctacggcaagctgaccctgaagttcatctgcaccaccggcaagctgcccgtgccctggcccaccctcgtgaccaccttcacctacggcgtgcagtgcttcagccgctaccccgaccacatgaagcagcacgacttcttcaagtccgccatgcccgaaggctacgtccaggagcgcaccatcttcttcaaggacgacggcaactacaagacccgcgccgaggtgaagttcgagggcgacaccctggtgaaccgcatcgagctgaagggcatcgacttcaaggaggacggcaacatcctggggcacaagctggagtacaactacaacagccacaacgtctatatcatggccgacaagcagaagaacggcatcaaggtgaacttcaagatccgccacaacatcgaggacggcagcgtgcagctcgccgaccactaccagcagaacacccccatcggcgacggccccgtgctgctgcccgacaaccactacctgagcacccagtccgccctgagcaaagaccccaacgagaagcgcgatcacatggtcctgctggagttcgtgaccgccgccgggatcactcacggcatggacgagctctacaagtaagcgatcgctgtacaccccccacccgcaaaactgtaggtggctcttaggagcacccacacccgttctagcccgaaagggcagaggtgggtcggcatggcatctccacctcctcgcggtccgacctgggcatccgaaggaggacgtcgtccactcggatggctaagggagagcctcgagtctagagggcccgcggttcgaaggtaagcctatccctaaccctctcctcggtctcgattctacgcgtaccggtcatcatcaccatcaccattgagtttaaacccgctgatcagcctcgactgtgccttctaagatccagacatgataagatacattgatgagtttggacaaaccacaactagaatgcagtgaaaaaaatgctttatttgtgaaatttgtgatgctattgctttatttgtaaccattataagctgcaataaacaagttaacaacaacaattgcattcattttatgtttcaggttcagggggaggtgtggggaggttttttaaagcaagtaaaacctctacaaatgtggtatggctgattatgatcagtcgacctgcaggcatgcaagcttggcgtaatcatggtcatagctgtttcctgtgtgaaattgttatccgctcacaattccacacaacatacgagccggaagcataaagtgtaaagcctggggtgcctaatgagtgagctaactcacattaattgcgttgcgctcactgcccgctttccagtcgggaaacctgtcgtgccagctgcattaatgaatcggccaacgcgcggggagaggcggtttgcgtattgggcgctcttccgcttcctcgctcactgactcgctgcgctcggtcgttcggctgcggcgagcggtatcagctcactcaaaggcggtaatacggttatccacagaatcaggggataacgcaggaaagaacatgtgagcaaaaggccagcaaaaggccaggaaccgtaaaaaggccgcgttgctggcgtttttccataggctccgcccccctgacgagcatcacaaaaatcgacgctcaagtcagaggtggcgaaacccgacaggactataaagataccaggcgtttccccctggaagctccctcg

# Supplementary Data 2

Sequence data for pMT plasmid expressing the recombinant Flock House Virus (FHV) RNA1, modified to express enhanced green fluorescent protein (eGFP) and *Drosophila melanogaster* *RPS13*

**>pMT-FHV RNA1 RPS13**

tggtgcccgatgtgactagctctttgctgcaggccgtcctatcctctggttccgataagagacccagaactccggccccccaccgcccaccgccacccccatacatatgtggtacgcaagtaagagtgcctgcgcatgccccatgtgccccaccaagagctttgcatcccatacaagtccccaaagtggagaaccgaaccaattcttcgcgggcagaacaaaagcttctgcacacgtctccactcgaatttggagccggccggcgtgtgcaaaagaggtgaatcgaacgaaagacccgtgtgtaaagccgcgtttccaaaatgtataaaaccgagagcatctggccaatgtgttttcgaaacaaataaaacagaaaagcgaacctaaacaatgactctaaaagttattcttggagaacaccagatcacccgaactgaattgttagtcgggattgcaaccgtatctgggtgcggtgccgtagtgtactgcatatccaagttctggggctatggggcaattgcgccctatcctcagagtggagggaaccgagttacacgcgcattgcaacgggctgtcattgacaaaacgaagaccccgatagagacacgtttctatccgcttgacagcctgcgtaccgtgacgcctaagcgtgtcgcagacaacgggcacgccgtttcaggggccgtacgtgatgccgcacgtcgtttgatcgacgagtccatcacggccgttggaggatccaaatttgaggtcaaccccaacccaaactcaagcactggactgcgaaaccatttccacttcgccgtcggtgatttggcacaagatttccgtaatgacacacctgcggatgatgccttcatcgtcggtgttgatgttgattattatgtcaccgagcctgatgtgcttttagagcacatgcgtccagtagtgttacacacctttaacccgaagaaagtgagcggttttgatgctgactcaccattcaccattaagaacaacttggttgaatataaggttagcggtggagcagcatgggtccatccagtttgggattggtgcgaagctggtgagtttatcgctagcagagtccgtaccagctggaaggagtggtttttacaactaccactgcgaatgattggtttggagaaagttggctatcataaaatccatcattgtagaccgtggactgattgtccagatcgtgcacttgtctacactataccgcaatatgtcatttggcggtttaattggattgataccgaactacacgtgcgaaaactgaaacggattgaataccaggacgaaaccaaacctggttggaacagattggagtatgtgaccgacaagaatgaactgctggtttccatcggtcgagaaggggagcatgctcagattactatcgagaaagaaaagttggatatgctctcgggattatccgccacccaatctgtcaacgctaggcttatcggtatgggacacaaggacccgcaatacacatccatgattgtccagtattatactggcaagaaggtagtgtcaccaattagtccaactgtgtataaacctacaatgccacgcgtccattggccagtaaccagtgacgcagatgtaccagaagtgagcgcgcgccaatacacactgcctatcgtgagtgactgtatgatgatgccaatgatcaagcgctgggaaacaatgtctgaatcaattgaacgtagggtgacttttgtcgccaatgataagaaaccaagcgacagaatcgccaaaatagccgaaacgtttgttaaattgatgaatgggccattcaaagatcttgaccctttgtcgattgaagaaacgattgaacggctgaataaaccgtcccaacaattacaacttagggcggttttcgaaatgattggagttgaacctcgtcaattgattgagtcgttcaacaagaacgaacctggaatgaaatctagccggataatatccggttttccagacatacttttcatcttgaaagtttccagatacaccttagcgtattcggatatagttctacatgccgaacacaatgaacattggtattaccccgggcggaacccgactgagatcgccgacggtgtttgtgagtttgttagtgactgtgatgctgaagtcatagaaactgacttctccaacctcgatggcagggtttccagctggatgcaaagaaacatcgcccaaaaggccatggttcaagcattccgcccagaatacagagatgagatcatttcattcatggacacgataatcaattgtccagctaaagctaaacgctttggtttccgatatgagcctggtgtaggcgttaaaagtggaagtccaacaaccacgccacataacacccaatacaatggatgtgtcgaatttacagctctgacctttgagcatcctgatgctgaacctgaagatttgttccgtttaatcggaccgaagtgcggtgatgatggtctttcccgggctatcattcaaaaatcaattaatcgcgctgccaagtgtttcggcctcgaactcaaagttgaacgatacaatccagagataggtctttgtttcctgtctcgtgtatttgtggacccgctcgcaactacgaccacaattcaagacccactgcgtactctgcgaaaactacatcttacaacaagagatccaacgataccattagctgatgcggcttgcgaccgtgtcgaaggctatctctgtaccgatgcgcttactccgttaatttcggattattgcaaaatggtactacgactctacgggcccactgcttcaactgagcaggtgagaaaccaacgtagaagccggaataaagagaagccctactggttgacttgtgacggatcatggccacagcatccgcaagacgcccatttgatgaagcaggttttaatcaaacgtacagccattgacgaagatcaggtcgatgcactcattgggcgttttgccgcaatgaaggatgtctgggagaaaattacacatgacagcgaggagagcgccgctgcgtgtacgtttgatgaagacggcgttgcgccgaactccgtggacgaatcgttaccaatgttaaacgatgccaagcaaactcgcgctaatccaggaacttcccgaccgcattcaaacggcggtggaagcagccatgggaatgagctaccaagacgcaccgaacaacgtgcgcagggacctcgacaacctgcacgcttgcctaaacaaggcaaaactaacggtaagtcggatggtaacatcactgctggagaaacccagcgtggtggcatacctagagggaaaggcccccgaggaggcaaaaccaacactcgaagaacgcctccgaaagctggagctcagccacagccttccaacaaccggaagtgaatgcatatggtgagcaagggcgaggagctgttcaccggggtggtgcccatcctggtcgagctggacggcgacgtgaacggccacaagttcagcgtgtccggcgagggcgagggcgatgccacctacggcaagctgaccctgaagttcatctgcaccaccggcaagctgcccgtgccctggcccaccctcgtgaccaccttcacctacggcgtgcagtgcttcagccgctaccccgaccacatgaagcagcacgacttcttcaagtccgccatgcccgaaggctacgtccaggagcgcaccatcttcttcaaggacgacggcaactacaagacccgcgccgaggtgaagttcgagggcgacaccctggtgaaccgcatcgagctgaagggcatcgacttcaaggaggacggcaacatcctggggcacaagctggagtacaactacaacagccacaacgtctatatcatggccgacaagcagaagaacggcatcaaggtgaacttcaagatccgccacaacatcgaggacggcagcgtgcagctcgccgaccactaccagcagaacacccccatcggcgacggccccgtgctgctgcccgacaaccactacctgagcacccagtccgccctgagcaaagaccccaacgagaagcgcgatcacatggtcctgctggagttcgtgaccgccgccgggatcactcacggcatggacgagctctacaagtaagcgatcgcgcagatgatgtcaaggagcagattaagaagctgggcaagaagggtctgactccctccaaaatcggcatcatcctgcgtgactcgcacggagttgcccaggtgcgtttcgtcaacggaaacaagatcctgcgcatcatgaagtcggtgggtctgaagcccgacattcccgaggatctgtaccacatgatcaagaaggccgtcgccatccgcaagcacttggagcgcaaccgcaaggacaaggacggcaagttccgtctgattctggtcgagtccaggatccaccgcctggcccgctactacaagaccaagagcgtcctgccccccaactggaaatacgagtcgagcactgcctccgccctggttgcctaagttcttgattcgctagttggtagtttgttttcaagtcttgcggggtcctacatgtacaccccccacccgcaaaactgtaggtggctcttaggagcacccacacccgttctagcccgaaagggcagaggtgggtcggcatggcatctccacctcctcgcggtccgacctgggcatccgaaggaggacgtcgtccactcggatggctaagggagagcctcgagtctagagggcccgcggttcgaaggtaagcctatccctaaccctctcctcggtctcgattctacgcgtaccggtcatcatcaccatcaccattgagtttaaacccgctgatcagcctcgactgtgccttctaagatccagacatgataagatacattgatgagtttggacaaaccacaactagaatgcagtgaaaaaaatgctttatttgtgaaatttgtgatgctattgctttatttgtaaccattataagctgcaataaacaagttaacaacaacaattgcattcattttatgtttcaggttcagggggaggtgtggggaggttttttaaagcaagtaaaacctctacaaatgtggtatggctgattatgatcagtcgacctgcaggcatgcaagcttggcgtaatcatggtcatagctgtttcctgtgtgaaattgttatccgctcacaattccacacaacatacgagccggaagcataaagtgtaaagcctggggtgcctaatgagtgagctaactcacattaattgcgttgcgctcactgcccgctttccagtcgggaaacctgtcgtgccagctgcattaatgaatcggccaacgcgcggggagaggcggtttgcgtattgggcgctcttccgcttcctcgctcactgactcgctgcgctcggtcgttcggctgcggcgagcggtatcagctcactcaaaggcggtaatacggttatccacagaatcaggggataacgcaggaaagaacatgtgagcaaaaggccagcaaaaggccaggaaccgtaaaaaggccgcgttgctggcgtttttccataggctccgcccccctgacgagcatcacaaaaatcgacgctcaagtcagaggtggcgaaacccgacaggactataaagataccaggcgtttccccctggaagctccctcg

# Supplementary Data 3

Sequence data for pMT plasmid expressing the recombinant Flock House Virus (FHV) RNA1, modified to express enhanced green fluorescent protein (eGFP) and *Drosophila melanogaster* *alpha COP*

**>pMT-FHV RNA1 alpha COP**

tggtgcccgatgtgactagctctttgctgcaggccgtcctatcctctggttccgataagagacccagaactccggccccccaccgcccaccgccacccccatacatatgtggtacgcaagtaagagtgcctgcgcatgccccatgtgccccaccaagagctttgcatcccatacaagtccccaaagtggagaaccgaaccaattcttcgcgggcagaacaaaagcttctgcacacgtctccactcgaatttggagccggccggcgtgtgcaaaagaggtgaatcgaacgaaagacccgtgtgtaaagccgcgtttccaaaatgtataaaaccgagagcatctggccaatgtgttttcgaaacaaataaaacagaaaagcgaacctaaacaatgactctaaaagttattcttggagaacaccagatcacccgaactgaattgttagtcgggattgcaaccgtatctgggtgcggtgccgtagtgtactgcatatccaagttctggggctatggggcaattgcgccctatcctcagagtggagggaaccgagttacacgcgcattgcaacgggctgtcattgacaaaacgaagaccccgatagagacacgtttctatccgcttgacagcctgcgtaccgtgacgcctaagcgtgtcgcagacaacgggcacgccgtttcaggggccgtacgtgatgccgcacgtcgtttgatcgacgagtccatcacggccgttggaggatccaaatttgaggtcaaccccaacccaaactcaagcactggactgcgaaaccatttccacttcgccgtcggtgatttggcacaagatttccgtaatgacacacctgcggatgatgccttcatcgtcggtgttgatgttgattattatgtcaccgagcctgatgtgcttttagagcacatgcgtccagtagtgttacacacctttaacccgaagaaagtgagcggttttgatgctgactcaccattcaccattaagaacaacttggttgaatataaggttagcggtggagcagcatgggtccatccagtttgggattggtgcgaagctggtgagtttatcgctagcagagtccgtaccagctggaaggagtggtttttacaactaccactgcgaatgattggtttggagaaagttggctatcataaaatccatcattgtagaccgtggactgattgtccagatcgtgcacttgtctacactataccgcaatatgtcatttggcggtttaattggattgataccgaactacacgtgcgaaaactgaaacggattgaataccaggacgaaaccaaacctggttggaacagattggagtatgtgaccgacaagaatgaactgctggtttccatcggtcgagaaggggagcatgctcagattactatcgagaaagaaaagttggatatgctctcgggattatccgccacccaatctgtcaacgctaggcttatcggtatgggacacaaggacccgcaatacacatccatgattgtccagtattatactggcaagaaggtagtgtcaccaattagtccaactgtgtataaacctacaatgccacgcgtccattggccagtaaccagtgacgcagatgtaccagaagtgagcgcgcgccaatacacactgcctatcgtgagtgactgtatgatgatgccaatgatcaagcgctgggaaacaatgtctgaatcaattgaacgtagggtgacttttgtcgccaatgataagaaaccaagcgacagaatcgccaaaatagccgaaacgtttgttaaattgatgaatgggccattcaaagatcttgaccctttgtcgattgaagaaacgattgaacggctgaataaaccgtcccaacaattacaacttagggcggttttcgaaatgattggagttgaacctcgtcaattgattgagtcgttcaacaagaacgaacctggaatgaaatctagccggataatatccggttttccagacatacttttcatcttgaaagtttccagatacaccttagcgtattcggatatagttctacatgccgaacacaatgaacattggtattaccccgggcggaacccgactgagatcgccgacggtgtttgtgagtttgttagtgactgtgatgctgaagtcatagaaactgacttctccaacctcgatggcagggtttccagctggatgcaaagaaacatcgcccaaaaggccatggttcaagcattccgcccagaatacagagatgagatcatttcattcatggacacgataatcaattgtccagctaaagctaaacgctttggtttccgatatgagcctggtgtaggcgttaaaagtggaagtccaacaaccacgccacataacacccaatacaatggatgtgtcgaatttacagctctgacctttgagcatcctgatgctgaacctgaagatttgttccgtttaatcggaccgaagtgcggtgatgatggtctttcccgggctatcattcaaaaatcaattaatcgcgctgccaagtgtttcggcctcgaactcaaagttgaacgatacaatccagagataggtctttgtttcctgtctcgtgtatttgtggacccgctcgcaactacgaccacaattcaagacccactgcgtactctgcgaaaactacatcttacaacaagagatccaacgataccattagctgatgcggcttgcgaccgtgtcgaaggctatctctgtaccgatgcgcttactccgttaatttcggattattgcaaaatggtactacgactctacgggcccactgcttcaactgagcaggtgagaaaccaacgtagaagccggaataaagagaagccctactggttgacttgtgacggatcatggccacagcatccgcaagacgcccatttgatgaagcaggttttaatcaaacgtacagccattgacgaagatcaggtcgatgcactcattgggcgttttgccgcaatgaaggatgtctgggagaaaattacacatgacagcgaggagagcgccgctgcgtgtacgtttgatgaagacggcgttgcgccgaactccgtggacgaatcgttaccaatgttaaacgatgccaagcaaactcgcgctaatccaggaacttcccgaccgcattcaaacggcggtggaagcagccatgggaatgagctaccaagacgcaccgaacaacgtgcgcagggacctcgacaacctgcacgcttgcctaaacaaggcaaaactaacggtaagtcggatggtaacatcactgctggagaaacccagcgtggtggcatacctagagggaaaggcccccgaggaggcaaaaccaacactcgaagaacgcctccgaaagctggagctcagccacagccttccaacaaccggaagtgaatgcatatggtgagcaagggcgaggagctgttcaccggggtggtgcccatcctggtcgagctggacggcgacgtgaacggccacaagttcagcgtgtccggcgagggcgagggcgatgccacctacggcaagctgaccctgaagttcatctgcaccaccggcaagctgcccgtgccctggcccaccctcgtgaccaccttcacctacggcgtgcagtgcttcagccgctaccccgaccacatgaagcagcacgacttcttcaagtccgccatgcccgaaggctacgtccaggagcgcaccatcttcttcaaggacgacggcaactacaagacccgcgccgaggtgaagttcgagggcgacaccctggtgaaccgcatcgagctgaagggcatcgacttcaaggaggacggcaacatcctggggcacaagctggagtacaactacaacagccacaacgtctatatcatggccgacaagcagaagaacggcatcaaggtgaacttcaagatccgccacaacatcgaggacggcagcgtgcagctcgccgaccactaccagcagaacacccccatcggcgacggccccgtgctgctgcccgacaaccactacctgagcacccagtccgccctgagcaaagaccccaacgagaagcgcgatcacatggtcctgctggagttcgtgaccgccgccgggatcactcacggcatggacgagctctacaagtaagcgatcgctgatcgccttgtgaagttgtggcgcatgaacgagtacaaggcttgggaggtcgacacctgtcgcggtcactacaacaacgtgtccagcgtgttgttccatcccaggcaagacctgattctctccaacggcgaagatcgcagtattcgcgtctgggacatgaccaagcgccagtgcctctttacattccggcgggacaacgagcgcttctggatacttacagcgcatccgaccctgaatctgtttgcagctggccacgatggaggtatggtggtctttaaactggagcgagagcgtcctgcatatgctgtccacgggaatattctctactacgtcaaggaacgtttccttcgcaaactggactttaccaccactaaggacactgtggttatgcagcttcgaccaggcaagtcgccggtgtacagcatgtcgtacaacccagcactgaacgccgtgctaatctgcacgcgcaccaacaacctggagaacagcacctacgattgtacaccccccacccgcaaaactgtaggtggctcttaggagcacccacacccgttctagcccgaaagggcagaggtgggtcggcatggcatctccacctcctcgcggtccgacctgggcatccgaaggaggacgtcgtccactcggatggctaagggagagcctcgagtctagagggcccgcggttcgaaggtaagcctatccctaaccctctcctcggtctcgattctacgcgtaccggtcatcatcaccatcaccattgagtttaaacccgctgatcagcctcgactgtgccttctaagatccagacatgataagatacattgatgagtttggacaaaccacaactagaatgcagtgaaaaaaatgctttatttgtgaaatttgtgatgctattgctttatttgtaaccattataagctgcaataaacaagttaacaacaacaattgcattcattttatgtttcaggttcagggggaggtgtggggaggttttttaaagcaagtaaaacctctacaaatgtggtatggctgattatgatcagtcgacctgcaggcatgcaagcttggcgtaatcatggtcatagctgtttcctgtgtgaaattgttatccgctcacaattccacacaacatacgagccggaagcataaagtgtaaagcctggggtgcctaatgagtgagctaactcacattaattgcgttgcgctcactgcccgctttccagtcgggaaacctgtcgtgccagctgcattaatgaatcggccaacgcgcggggagaggcggtttgcgtattgggcgctcttccgcttcctcgctcactgactcgctgcgctcggtcgttcggctgcggcgagcggtatcagctcactcaaaggcggtaatacggttatccacagaatcaggggataacgcaggaaagaacatgtgagcaaaaggccagcaaaaggccaggaaccgtaaaaaggccgcgttgctggcgtttttccataggctccgcccccctgacgagcatcacaaaaatcgacgctcaagtcagaggtggcgaaacccgacaggactataaagataccaggcgtttccccctggaagctccctcg

# Supplementary Data 4

Sequence data for pMT plasmid expressing the recombinant Flock House Virus (FHV) RNA1, modified to express enhanced green fluorescent protein (eGFP) and *Drosophila melanogaster* *Vha26*

**>pMT-FHV RNA1 Vha26**

tggtgcccgatgtgactagctctttgctgcaggccgtcctatcctctggttccgataagagacccagaactccggccccccaccgcccaccgccacccccatacatatgtggtacgcaagtaagagtgcctgcgcatgccccatgtgccccaccaagagctttgcatcccatacaagtccccaaagtggagaaccgaaccaattcttcgcgggcagaacaaaagcttctgcacacgtctccactcgaatttggagccggccggcgtgtgcaaaagaggtgaatcgaacgaaagacccgtgtgtaaagccgcgtttccaaaatgtataaaaccgagagcatctggccaatgtgttttcgaaacaaataaaacagaaaagcgaacctaaacaatgactctaaaagttattcttggagaacaccagatcacccgaactgaattgttagtcgggattgcaaccgtatctgggtgcggtgccgtagtgtactgcatatccaagttctggggctatggggcaattgcgccctatcctcagagtggagggaaccgagttacacgcgcattgcaacgggctgtcattgacaaaacgaagaccccgatagagacacgtttctatccgcttgacagcctgcgtaccgtgacgcctaagcgtgtcgcagacaacgggcacgccgtttcaggggccgtacgtgatgccgcacgtcgtttgatcgacgagtccatcacggccgttggaggatccaaatttgaggtcaaccccaacccaaactcaagcactggactgcgaaaccatttccacttcgccgtcggtgatttggcacaagatttccgtaatgacacacctgcggatgatgccttcatcgtcggtgttgatgttgattattatgtcaccgagcctgatgtgcttttagagcacatgcgtccagtagtgttacacacctttaacccgaagaaagtgagcggttttgatgctgactcaccattcaccattaagaacaacttggttgaatataaggttagcggtggagcagcatgggtccatccagtttgggattggtgcgaagctggtgagtttatcgctagcagagtccgtaccagctggaaggagtggtttttacaactaccactgcgaatgattggtttggagaaagttggctatcataaaatccatcattgtagaccgtggactgattgtccagatcgtgcacttgtctacactataccgcaatatgtcatttggcggtttaattggattgataccgaactacacgtgcgaaaactgaaacggattgaataccaggacgaaaccaaacctggttggaacagattggagtatgtgaccgacaagaatgaactgctggtttccatcggtcgagaaggggagcatgctcagattactatcgagaaagaaaagttggatatgctctcgggattatccgccacccaatctgtcaacgctaggcttatcggtatgggacacaaggacccgcaatacacatccatgattgtccagtattatactggcaagaaggtagtgtcaccaattagtccaactgtgtataaacctacaatgccacgcgtccattggccagtaaccagtgacgcagatgtaccagaagtgagcgcgcgccaatacacactgcctatcgtgagtgactgtatgatgatgccaatgatcaagcgctgggaaacaatgtctgaatcaattgaacgtagggtgacttttgtcgccaatgataagaaaccaagcgacagaatcgccaaaatagccgaaacgtttgttaaattgatgaatgggccattcaaagatcttgaccctttgtcgattgaagaaacgattgaacggctgaataaaccgtcccaacaattacaacttagggcggttttcgaaatgattggagttgaacctcgtcaattgattgagtcgttcaacaagaacgaacctggaatgaaatctagccggataatatccggttttccagacatacttttcatcttgaaagtttccagatacaccttagcgtattcggatatagttctacatgccgaacacaatgaacattggtattaccccgggcggaacccgactgagatcgccgacggtgtttgtgagtttgttagtgactgtgatgctgaagtcatagaaactgacttctccaacctcgatggcagggtttccagctggatgcaaagaaacatcgcccaaaaggccatggttcaagcattccgcccagaatacagagatgagatcatttcattcatggacacgataatcaattgtccagctaaagctaaacgctttggtttccgatatgagcctggtgtaggcgttaaaagtggaagtccaacaaccacgccacataacacccaatacaatggatgtgtcgaatttacagctctgacctttgagcatcctgatgctgaacctgaagatttgttccgtttaatcggaccgaagtgcggtgatgatggtctttcccgggctatcattcaaaaatcaattaatcgcgctgccaagtgtttcggcctcgaactcaaagttgaacgatacaatccagagataggtctttgtttcctgtctcgtgtatttgtggacccgctcgcaactacgaccacaattcaagacccactgcgtactctgcgaaaactacatcttacaacaagagatccaacgataccattagctgatgcggcttgcgaccgtgtcgaaggctatctctgtaccgatgcgcttactccgttaatttcggattattgcaaaatggtactacgactctacgggcccactgcttcaactgagcaggtgagaaaccaacgtagaagccggaataaagagaagccctactggttgacttgtgacggatcatggccacagcatccgcaagacgcccatttgatgaagcaggttttaatcaaacgtacagccattgacgaagatcaggtcgatgcactcattgggcgttttgccgcaatgaaggatgtctgggagaaaattacacatgacagcgaggagagcgccgctgcgtgtacgtttgatgaagacggcgttgcgccgaactccgtggacgaatcgttaccaatgttaaacgatgccaagcaaactcgcgctaatccaggaacttcccgaccgcattcaaacggcggtggaagcagccatgggaatgagctaccaagacgcaccgaacaacgtgcgcagggacctcgacaacctgcacgcttgcctaaacaaggcaaaactaacggtaagtcggatggtaacatcactgctggagaaacccagcgtggtggcatacctagagggaaaggcccccgaggaggcaaaaccaacactcgaagaacgcctccgaaagctggagctcagccacagccttccaacaaccggaagtgaatgcatatggtgagcaagggcgaggagctgttcaccggggtggtgcccatcctggtcgagctggacggcgacgtgaacggccacaagttcagcgtgtccggcgagggcgagggcgatgccacctacggcaagctgaccctgaagttcatctgcaccaccggcaagctgcccgtgccctggcccaccctcgtgaccaccttcacctacggcgtgcagtgcttcagccgctaccccgaccacatgaagcagcacgacttcttcaagtccgccatgcccgaaggctacgtccaggagcgcaccatcttcttcaaggacgacggcaactacaagacccgcgccgaggtgaagttcgagggcgacaccctggtgaaccgcatcgagctgaagggcatcgacttcaaggaggacggcaacatcctggggcacaagctggagtacaactacaacagccacaacgtctatatcatggccgacaagcagaagaacggcatcaaggtgaacttcaagatccgccacaacatcgaggacggcagcgtgcagctcgccgaccactaccagcagaacacccccatcggcgacggccccgtgctgctgcccgacaaccactacctgagcacccagtccgccctgagcaaagaccccaacgagaagcgcgatcacatggtcctgctggagttcgtgaccgccgccgggatcactcacggcatggacgagctctacaagtaagcgatcgcagcaccgaaatggacctaaacccccgatttcgcttcttcgagggcaacggacgcttgtgcaactgccactggctcaacgaaagccccgaaaatcatcaatgtctgttgttgttgagataccgagagtagagaatacacactgcttagcacgcgacacttaatacccattcattacacatgcaccacgacgatgaagtttgccaagtagctaagttgttgacctgaccatcaagtgcagctttcacaccctcatataactacttaaagaaaatatagaaaaatggaaattagttttgcaatttaggccactgccgaactgccaccgtttccacctgacgtgcgccatcatatcaggctctaaaaatcaacacaccatgttcaaacacacgactagcatacaggagcaggagctacagtaaatttgaaccttgtattcgcatgttcgccaatgtacaccccccacccgcaaaactgtaggtggctcttaggagcacccacacccgttctagcccgaaagggcagaggtgggtcggcatggcatctccacctcctcgcggtccgacctgggcatccgaaggaggacgtcgtccactcggatggctaagggagagcctcgagtctagagggcccgcggttcgaaggtaagcctatccctaaccctctcctcggtctcgattctacgcgtaccggtcatcatcaccatcaccattgagtttaaacccgctgatcagcctcgactgtgccttctaagatccagacatgataagatacattgatgagtttggacaaaccacaactagaatgcagtgaaaaaaatgctttatttgtgaaatttgtgatgctattgctttatttgtaaccattataagctgcaataaacaagttaacaacaacaattgcattcattttatgtttcaggttcagggggaggtgtggggaggttttttaaagcaagtaaaacctctacaaatgtggtatggctgattatgatcagtcgacctgcaggcatgcaagcttggcgtaatcatggtcatagctgtttcctgtgtgaaattgttatccgctcacaattccacacaacatacgagccggaagcataaagtgtaaagcctggggtgcctaatgagtgagctaactcacattaattgcgttgcgctcactgcccgctttccagtcgggaaacctgtcgtgccagctgcattaatgaatcggccaacgcgcggggagaggcggtttgcgtattgggcgctcttccgcttcctcgctcactgactcgctgcgctcggtcgttcggctgcggcgagcggtatcagctcactcaaaggcggtaatacggttatccacagaatcaggggataacgcaggaaagaacatgtgagcaaaaggccagcaaaaggccaggaaccgtaaaaaggccgcgttgctggcgtttttccataggctccgcccccctgacgagcatcacaaaaatcgacgctcaagtcagaggtggcgaaacccgacaggactataaagataccaggcgtttccccctggaagctccctcg

# Supplementary Data 5

Alignment of RNA directed RNA polymerase (RdRp) amino acid sequence from recombinant FHV with an RdRp amino acid sequence (Q66929 -RDRP_FHV) from UniProt database using CLUSTAL OMEGA (1.2.4)

sp|Q66929|RDRP_FHV MTLKVILGEHQITRTELLVGIATVSGCGAVVYCISKFWGYGAIAPYPQSGGNRVTRALQR

pMT_FHV_RNA-directed mtlkvilgehqitrtellvgiatvsgcgavvyciskfwgygaiapypqsggnrvtralqr

************************************************************

sp|Q66929|RDRP_FHV AVIDKTKTPIETRFYPLDSLRTVTPKRVADNGHAVSGAVRDAARRLIDESITAVGGSKFE

pMT_FHV_RNA-directed avidktktpietrfypldslrtvtpkrvadnghavsgavrdaarrlidesitavggskfe

************************************************************

sp|Q66929|RDRP_FHV VNPNPNSSTGLRNHFHFAVGDLAQDFRNDTPADDAFIVGVDVDYYVTEPDVLLEHMRPVV

pMT_FHV_RNA-directed vnpnpnsstglrnhfhfavgdlaqdfrndtpaddafivgvdvdyyvtepdvllehmrpvv

************************************************************

sp|Q66929|RDRP_FHV LHTFNPKKVSGFDADSPFTIKNNLVEYKVSGGAAWVHPVWDWCEAGEFIASRVRTSWKEW

pMT_FHV_RNA-directed lhtfnpkkvsgfdadspftiknnlveykvsggaawvhpvwdwceagefiasrvrtswkew

************************************************************

sp|Q66929|RDRP_FHV FLQLPLRMIGLEKVGYHKIHHCRPWTDCPDRALVYTIPQYVIWRFNWIDTELHVRKLKRI

pMT_FHV_RNA-directed flqlplrmiglekvgyhkihhcrpwtdcpdralvytipqyviwrfnwidtelhvrklkri

************************************************************

sp|Q66929|RDRP_FHV EYQDETKPGWNRLEYVTDKNELLVSIGREGEHAQITIEKEKLDMLSGLSATQSVNARLIG

pMT_FHV_RNA-directed eyqdetkpgwnrleyvtdknellvsigregehaqitiekekldmlsglsatqsvnarlig

************************************************************

sp|Q66929|RDRP_FHV MGHKDPQYTSMIVQYYTGKKVVSPISPTVYKPTMPRVHWPVTSDADVPEVSARQYTLPIV

pMT_FHV_RNA-directed mghkdpqytsmivqyytgkkvvspisptvykptmprvhwpvtsdadvpevsarqytlpiv

************************************************************

sp|Q66929|RDRP_FHV SDCMMMPMIKRWETMSESIERRVTFVANDKKPSDRIAKIAETFVKLMNGPFKDLDPLSIE

pMT_FHV_RNA-directed sdcmmmpmikrwetmsesierrvtfvandkkpsdriakiaetfvklmngpfkdldplsie

************************************************************

sp|Q66929|RDRP_FHV ETIERLNKPSQQLQLRAVFEMIGVKPRQLIESFNKNEPGMKSSRIISGFPDILFILKVSR

pMT_FHV_RNA-directed etierlnkpsqqlqlravfemigveprqliesfnknepgmkssriisgfpdilfilkvsr

************************:***********************************

sp|Q66929|RDRP_FHV YTLAYSDIVLHAEHNEHWYYPGRNPTEIADGVCEFVSDCDAEVIETDFSNLDGRVSSWMQ

pMT_FHV_RNA-directed ytlaysdivlhaehnehwyypgrnpteiadgvcefvsdcdaevietdfsnldgrvsswmq

************************************************************

sp|Q66929|RDRP_FHV RNIAQKAMVQAFRPEYRDEIISFMDTIINCPAKAKRFGFRYEPGVGVKSGSPTTTPHNTQ

pMT_FHV_RNA-directed rniaqkamvqafrpeyrdeiisfmdtiincpakakrfgfryepgvgvksgsptttphntq

************************************************************

sp|Q66929|RDRP_FHV YNGCVEFTALTFEHPDAEPEDLFRLIGPKCGDDGLSRAIIQKSINRAAKCFGLELKVERY

pMT_FHV_RNA-directed yngcveftaltfehpdaepedlfrligpkcgddglsraiiqksinraakcfglelkvery

************************************************************

sp|Q66929|RDRP_FHV NPEIGLCFLSRVFVDPLATTTTIQDPLRTLRKLHLTTRDPTIPLADAACDRVEGYLCTDA

pMT_FHV_RNA-directed npeiglcflsrvfvdplattttiqdplrtlrklhlttrdptipladaacdrvegylctda

************************************************************

sp|Q66929|RDRP_FHV LTPLISDYCKMVLRLYGPTASTEQVRNQRRSRNKEKPYWLTCDGSWPQHPQDAHLMKQVL

pMT_FHV_RNA-directed ltplisdyckmvlrlygptasteqvrnqrrsrnkekpywltcdgswpqhpqdahlmkqvl

************************************************************

sp|Q66929|RDRP_FHV IKRTAIDEDQVDALIGRFAAMKDVWEKITHDSEESAAACTFDEDGVAPNSVDESLPMLND

pMT_FHV_RNA-directed ikrtaidedqvdaligrfaamkdvwekithdseesaaactfdedgvapnsvdeslpmlnd

************************************************************

sp|Q66929|RDRP_FHV AKQTRANPGTSRPHSNGGGSSHGNELPRRTEQRAQGPRQPARLPKQGKTNGKSDGNITAG

pMT_FHV_RNA-directed akqtranpgtsrphsngggsshgnelprrteqraqgprqparlpkqgktngksdgnitag

************************************************************

sp|Q66929|RDRP_FHV ETQRGGIPRGKGPRGGKTNTRRTPPKAGAQPQPSNNRK

pMT_FHV_RNA-directed etqrggiprgkgprggktntrrtppkagaqpqpsnnrk

**************************************

# Supplementary Data 6

Alignment of B2 Protein amino acid sequence from recombinant FHV with a B2 Protein amino acid sequence (P68831-B2_FHV) from UniProt database using CLUSTAL OMEGA (1.2.4)

sp|P68831|B2_FHV MPSKLALIQELPDRIQTAVEAAMGMSYQDAPNNVRRDLDNLHACLNKAKLTVSRMVTSLL

pMT_FHV_B2 mpsklaliqelpdriqtaveaamgmsyqdapnnvrrdldnlhaclnkakltvsrmvtsll

************************************************************

sp|P68831|B2_FHV EKPSVVAYLEGKAPEEAKPTLEERLRKLELSHSLPTTGSDPPPAKL

pMT_FHV_B2 ekpsvvaylegkapeeakptleerlrklelshslpttgseciw---

***************************************:

# Supplementary Data 7


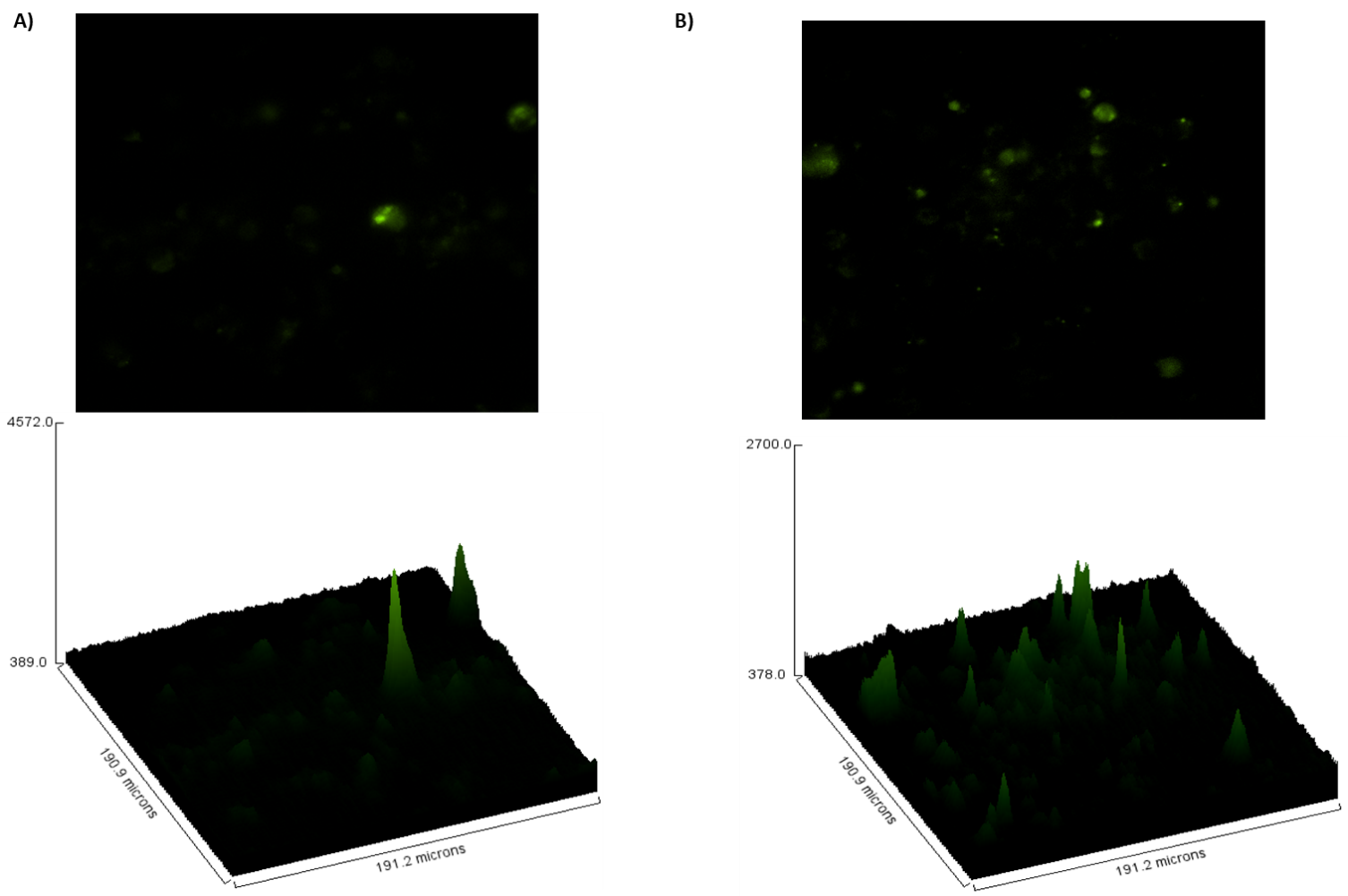


**Supplementary Figure 1:** Fluorescence of eGFP in S2 cells following expression of the recombinant FHV, engineered to express eGFP. A) eGFP fluorescence in S2 cells at 72 h after activation of the recombinant plasmid (pMT-FHV RNA1 eGFP) to express the engineered FHV. B) eGFP fluorescence in S2 cells at 72 h following infection of virus-free S2 cells with an unpurified virus supernatant, containing the recombinant FHV

# Supplementary Data 8

**Supplementary Table 1**: Primers used for detecting the virus in the inoculums

| Target | Primer sequence (5′-3′) | Product size (bp) |
| --- | --- | --- |
| FHV RNA1 | Forward: GCCTGGTGTAGGCGTTAAAA  Reverse: CAGGATGCTCAAAGGTCAGAG | 101bp |

**Supplementary Figure 2:** qRT-PCR confirmation that the viral titer was the same between the gene- targeting and non-gene targeting FHV inoculums for the *in vitro* and *in vivo* bioassays. Bars represent the mean±standard error of the cycle threshold value (Ct-value). Different letters indicate statistically significant differences (*p*<0.05).
